# Supplementary material for: Effects of Message Framing, Sender Authority, and Recipients’ Self-Reported Trait Autonomy on Endorsement of Health and Safety Measures during the Early COVID-19 Pandemic
Source: Int J Environ Res Public Health. 2021 Jul 21;18(15):7740. doi: 10.3390/ijerph18157740 (PMC8345362; doi:10.3390/ijerph18157740)
Supplement: Supplementary file 1 [file ijerph-18-07740-s001.zip › ijerph-1275970-supple.pdf]

Supplementary Materials

Table S1. Trait autonomy items and item source.

item text	inverted	item source
If I get into trouble, it is my own fault even if someone else told me to do it.	-	Black, J. E. (2016). An introduction to the moral agency scale. *Social Psychology*. https://doi.org/10.1027/1864-9335/a000284
I make up my own mind about doing good or bad things.	-	
I am just as at fault for breaking the rules when no one knows as when everyone knows.	-	
I am the one responsible for my own behavior, good and bad.	-	
I feel responsible for the consequences of my actions.	-	
Most of the time I can tell how my actions are going to affect others	-	
In most cases, I can make my own decisions about what is right or wrong in a situation.	-	
If I feel pressured into doing something, I’m not as responsible as when I decide on my own	yes	
No one can make me do something I know to be wrong.	-	
My actions in most situations are based on what other people tell me is the right thing to do.	yes	
I find it hard to make decisions on my own.	yes	Becker, P. (1989). Der Trierer Persönlichkeitsfragebogen (TPF). *Handanweisung.* Göttingen: Hogrefe.
When I have a difficult problem to solve, I ask someone to help me.	yes	
I lean on stronger people.	yes	
I like to make important decisions alone.	-	
I want to take responsibility for my life alone.	-	
I like to go my own way.	-	
It is my feeling that if everyone else in a group is behaving in a certain manner, this must be the proper way to behave.	yes	Laux, L. & Renner, K.-H. (2002). Self-Monitoring und Authentizität: Die verkannten Selbstdarsteller. *Zeitschrift für Differentielle und Diagnostische Psychologie*, 23, 129-148. https://doi.org/10.1024//0170-1789.23.2.129
When I am uncertain how to act in a social situation, I look to the behavior of others for cues.	yes	
I try to pay attention to the reactions of others to my behavior in order to avoid being out of place.	yes	
The slightest look of disapproval in the eyes of a person with whom I am interacting is enough to make me change my approach.	yes	
It’s important to me to fit in to the group I’m with.	yes	
My behavior often depends on how I feel others wish me to behave.	yes	

Exploratory Analysis on socio-demographics

To examine the possible influence of socio-demographic variables, we calculated a hierarchical regression. We calculated a regression using age, gender and education as predictors for the absolute shift. Then conducted a hierarchical regression: in the first step we used the significant predictors and then step wise added the predictors autonomy, sender and message. We first conducted this analysis for the absolute pre-post differences across all seven items (Table S2) and then only for item 5 (Table S3).

Table S2. Hierarchical regression results using absolute pre-post differences (averaged across all seven items) as the criterion, with correction of ceiling effects.

		ΔR^2	*b*	*b* 95% *CI*	*p*
step 0		0.01			0.19
	constant		0.63	[0.17, 1.10]	<.01
	age		0.001	[-0.001, 0.005]	0.24
	gender		-0,10	[-0.21, -0.001]	**0.05**
	education		0.01	[-0.03, 0.05]	0.68
step 1		0.01			0.07
	constant		0.76	[0.62, 0.91	<.001
	gender		-0.09	[-0.19, 0.01]	0.07
step 2		0.03			<.001
	constant		1.05	[0.49, 1.61]	<.001
	gender		-0.09	[-0.19, 0.00]	0.07
	autonomy		-0.07	[-0.23, 0.07]	.30
step 3		0.01			.40
	constant		1.06	[0.49, 1.62]	<0.01
	gender		-0.09	[-0.00,-0.00]	0.07
	autonomy		-0.07	[-0.23, 0.07]	0.31
	sender (social worker)		0.01	[-0.16, 0.11]	0.81
	message (moral)		-0.04	[-0.16, 0.07]	0.48
	message (control)		-0.00	[-0.12. 0.12]	0.98

Note. b represents unstandardized regression weights. Square brackets are used to enclose the lower and upper limits of a confidence interval.

Table S3. Hierarchical regression results using absolute pre-post differences for item 5 as the criterion (the one item that showed no ceiling effect).

		ΔR^2	*b*	*b 95% CI*	*p*
step 0		0.04			<0.001
	constant		0.66	[0.02, 1.31]	0.04
	age		-0.01	[-0.01, -0.00]	**<0.001**
	gender		-0,18	[-0.32, -0.04]	**0.02**
	education		0.05	[-0.01, 0.12]	0.08
step 1		0.03			0.07
	constant		1.1<br>9	[0.96, 1.43]	**<0.001**
	age		-0.01	[-0.01, -0.00]	**<0.001**
	gender		-0.19	[-0.34, -0.04]	**0.01**
step 2		0.04			<0.001
	constant		2.0<br>5	[1.26, 2.84]	<0.001
	age		-0.01	[-0.01, -0.00]	**<0.001**
	gender		-0.19	[-0.34, -0.04]	**0.01**
	autonomy		-0.24	[-0.46, -0.02]	**0.03**
step 3		0.04			<0.001
	constant		2.12	[1.31, 2.92]	<0.001
	age		-0.01	[-0.01, -0.00]	**<0.01**
	gender		-0.19	[-0.34, -0.04]	**<0.01**
	autonomy		-0.25	[-0.47, -0.03]	**0.02**
	sender (social worker)		-0.01	[-0.16, 0.12]	0.84
	message (moral)		-0.09	[-0.26, 0.08]	0.28
	message (control)		-0.05	[-0.23. 0.11]	0.49

Note. b represents unstandardized regression weights. Square brackets are used to enclose the lower and upper limits of a confidence interval.

Table S4. ANOVA results on single item level, without and with correction of ceiling effect.

	F(df, dfd)	*p*	*η2*
item 1 - without correction of ceiling effect			
message	F(2,701) = 0.75	0.47	0.00
sender	F(1,701) = 2.25	0.13	0
measurement	F(1,701) = 3.25	0.07	0
message:sender	F(2,701) = 0.15	0.86	0
message:measurement	F(2,701) = 0.27	0.76	0
sender:measurement	F(1,701) = 0.42	0.52	0
message:sender:measurement	F(2,701) = 0.95	0.39	0
item 1 – with correction of ceiling effect			
message	F(2,295) = 2.46	0.09	0
sender	F(1,295) = 0.03	0.86	0
measurement	**F(1,295) = 16.77**	**0.00**	**0**
message:sender	F(2,295) = 0.03	0.97	0
message:measurement	F(2,295) = 0.61	0.54	0
sender:measurement	F(1,295) = 3.79	0.05	0
message:sender:measurement	F(2,295) = 0.02	0.98	0
item 2 - without correction of ceiling effect			
message	F(2,701) = 0.03	0.97	0
sender	F(1,701) = 0.53	0.47	0
measurement	**F(1,701) = 9.44**	**0.00**	**0**
message:sender	F(2,701) = 0.69	0.50	0
message:measurement	F(2,701) = 2.24	0.11	0
sender:measurement	F(1,701) = 0.41	0.52	0
message:sender:measurement	F(2,701) = 0.84	0.43	0
item 2 –with correction of ceiling effect			
message	F(2,217) = 1.89	0.15	0
sender	F(1,217) = 0.02	0.89	0
measurement	**F(1,217) = 45.61**	**0.00**	**0.05**
message:sender	F(2,217) = 0.73	0.48	0
message:measurement	F(2,217) = 1.16	0.32	0
sender:measurement	F(1,217) = 0.01	0.92	0
message:sender:measurement	F(2,217) = 1.89	0.15	0
item 3 - without correction of ceiling effect			
message	F(2,701) = 2.22	0.11	0
sender	F(1,701) = 0.17	0.68	0
measurement	**F(1,701) = 20.38**	**0.00**	**0.01**
message:sender	F(2,701) = 2.18	0.11	0
message:measurement	F(2,701) = 0.76	0.47	0
sender:measurement	F(1,701) = 1.11	0.29	0
message:sender:measurement	**F(2,701) = 3.29**	**0.04**	**0**
item 3 – with correction of ceiling effect			
message	F(2,147) = 1.66	0.19	0.02
sender	F(1,147) = 0.28	0.60	0
measurement	**F(1,147) = 9.44**	**0.00**	**0.02**
message:sender	F(2,147) = 0.15	0.86	0
message:measurement	F(2,147) = 0.74	0.48	0
sender:measurement	F(1,147) = 0.11	0.74	0
message:sender:measurement	F(2,147) = 1.42	0.24	0
item 4 - without correction of ceiling effect			
message	F(2,701) = 1.71	0.18	0
sender	F(1,701) = 0.56	0.46	0
measurement	F(1,701) = 0.25	0.62	0
message:sender	F(2,701) = 1.91	0.15	0
message:measurement	F(2,701) = 0.15	0.86	0
sender:measurement	**F(1,701) = 4.05**	**0.04**	**0**
message:sender:measurement	F(2,701) = 1.06	0.34	0

	F(df, dfd)	*p*	*η2*
item 4 – with correction of ceiling effect			
message	F(2,323) = 2.82	0.06	0
sender	F(1,323) = 3.08	0.08	0.01
measurement	**F(1,323) = 21.80**	**0.00**	**0**
message:sender	F(2,323) = 1.56	0.21	0.01
message:measurement	F(2,323) = 0.53	0.59	0
sender:measurement	F(1,323) = 5.02	0.03*	0
message:sender:measurement	F(2,323) = 0.54	0.58	0
item 5 - without correction of ceiling effect			
message	F(2,701) = 2.45	0.09	0.01
sender	F(1,701) = 0.12	0.74	0
measurement	**F(1,701) = 220.62**	**0.00**	**0.03**
message:sender	F(2,701) = 1.14	0.32	0
message:measurement	F(2,701) = 0.28	0.76	0
sender:measurement	F(1,701) = 1.49	0.22	0
message:sender:measurement	F(2,701) = 1.47	0.23	0
item 5 – with correction of ceiling effect			
message	F(2,595) = 0.79	0.45	0.00
sender	F(1,595) = 0.85	0.36	0.00
measurement	**F(1,595) = 285.08**	**0.00**	**0.08**
message:sender	F(2,595) = 1.21	0.30	0.01
message:measurement	F(2,595) = 1.07	0.34	0
sender:measurement	F(1,595) = 0.96	0.33	0
message:sender:measurement	F(2,595) = 2.05	0.13	0
item 6 - without correction of ceiling effect			
message	F(2,701) = 0.32	0.72	0
sender	F(1,701) = 2.77	0.10	0
measurement	F(1,701) = 0.20	0.65	0
message:sender	F(2,701) = 1.35	0.26	0
message:measurement	F(2,701) = 1.83	0.16	0
sender:measurement	F(1,701) = 0.01	0.92	0
message:sender:measurement	F(2,701) = 0.48	0.62	0
item 6 – with correction of ceiling effect			
message	F(2,343) = 0.01	0.99	0.00
sender	F(1,343) = 0.23	0.63	0
measurement	**F(1,343) = 14.09**	**0.00**	**0**
message:sender	F(2,343) = 0.19	0.83	0
message:measurement	**F(2,343) = 3.59**	**0.03**	**0**
sender:measurement	F(1,343) = 0.75	0.39	0
message:sender:measurement	F(2,343) = 0.50	0.61	0
item 7 - without correction of ceiling effect			
message	F(2,701) = 0.07	0.93	0
sender	F(1,701) = 0.33	0.57	0
measurement	F(1,701) = 0.54	0.46	0
message:sender	F(2,701) = 0.43	0.65	0
message:measurement	F(2,701) = 0.30	0.74	0
sender:measurement	F(1,701) = 0.49	0.49	0
message:sender:measurement	F(2,701) = 1.67	0.19	0
item 7 – with correction of ceiling effect			
message	F(2,219) = 0.05	0.95	0.00
sender	**F(1,219) = 4.33**	**0.04**	**0.01**
measurement	**F(1,219) = 26.95**	**0.00**	**0.03**
message:sender	F(2,219) = 0.22	0.80	0
message:measurement	F(2,219) = 0.06	0.95	0
sender:measurement	F(1,219) = 0.01	0.91	0
message:sender:measurement	*F(*2,219) = 0.18	0.83	0

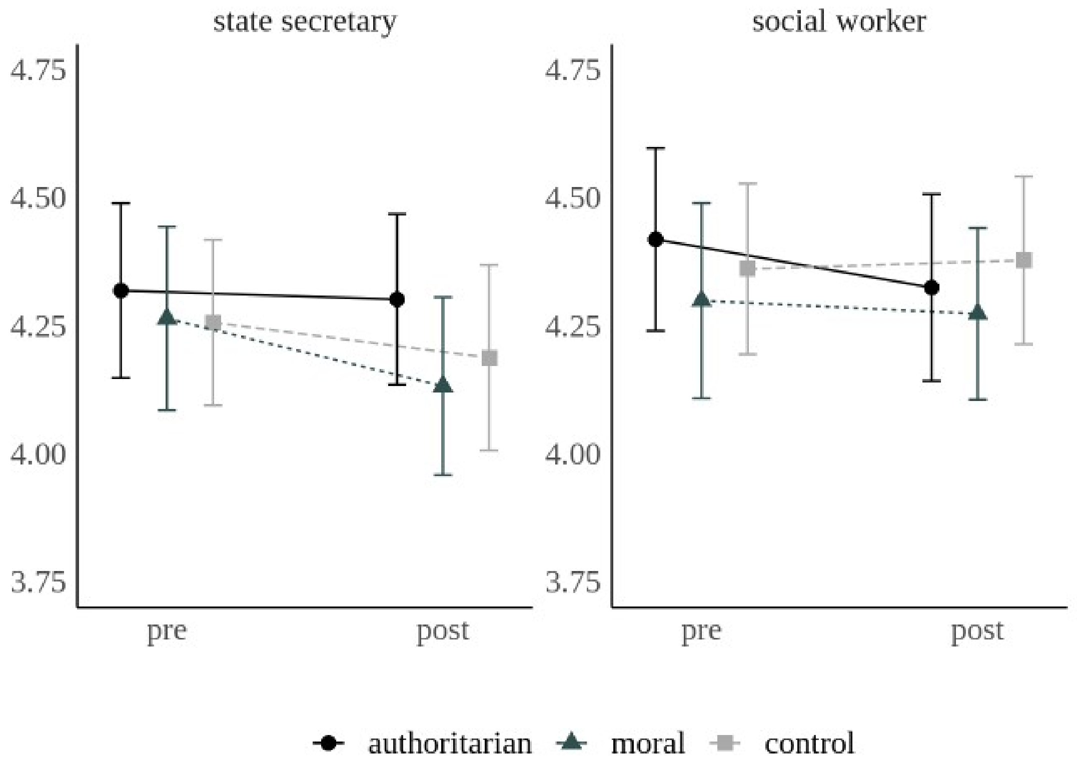

Item 1: I reduce contacts to other people outside the apartment to an absolute minimum. (*without correction of ceiling effect*)

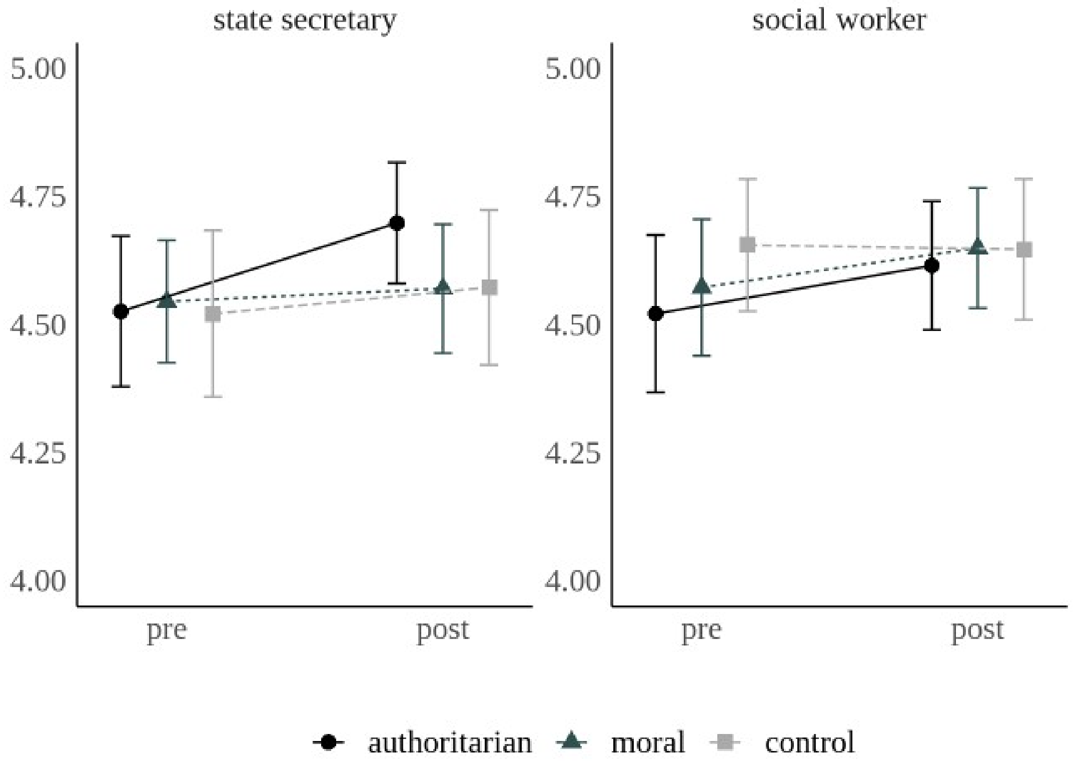

Item 2: I keep a minimum distance of 1.5 meter to other people in public wherever possible. (*without correction of ceiling effect*)

Figure S1. Mean ratings (95% CI) in response to the single items, without correction of the ceiling effects

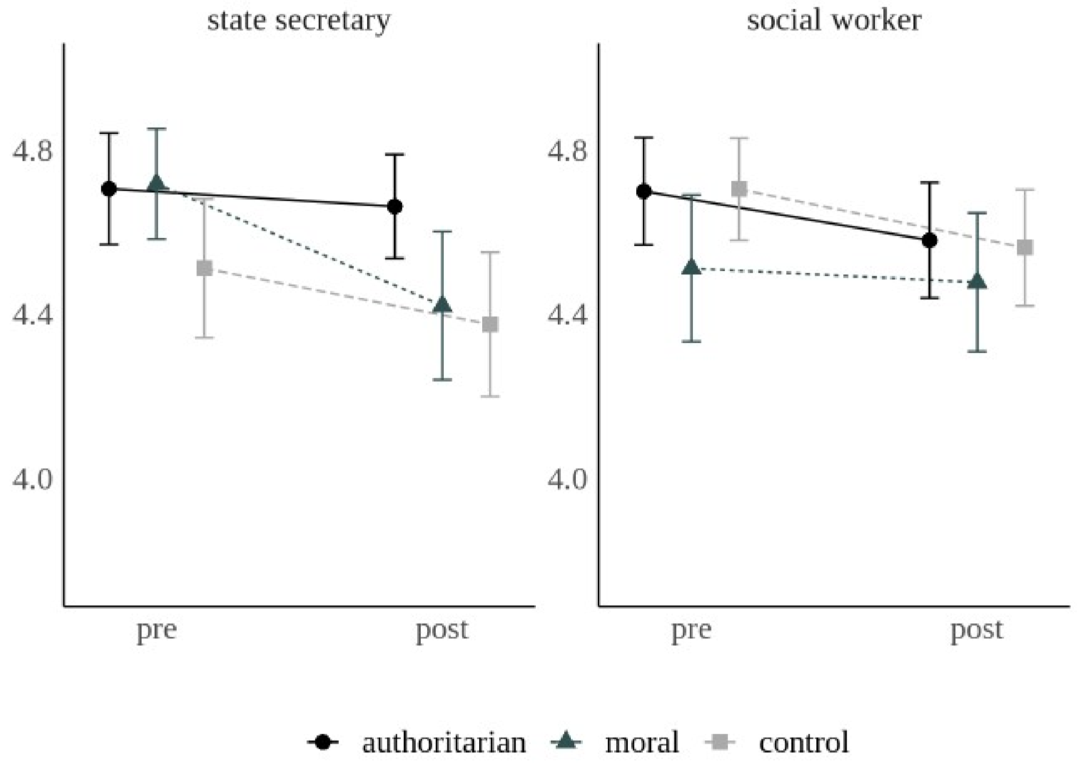

Item 3: I only spend time in public alone, with people of my household, or with one other person. (*without correction of ceiling effect*)

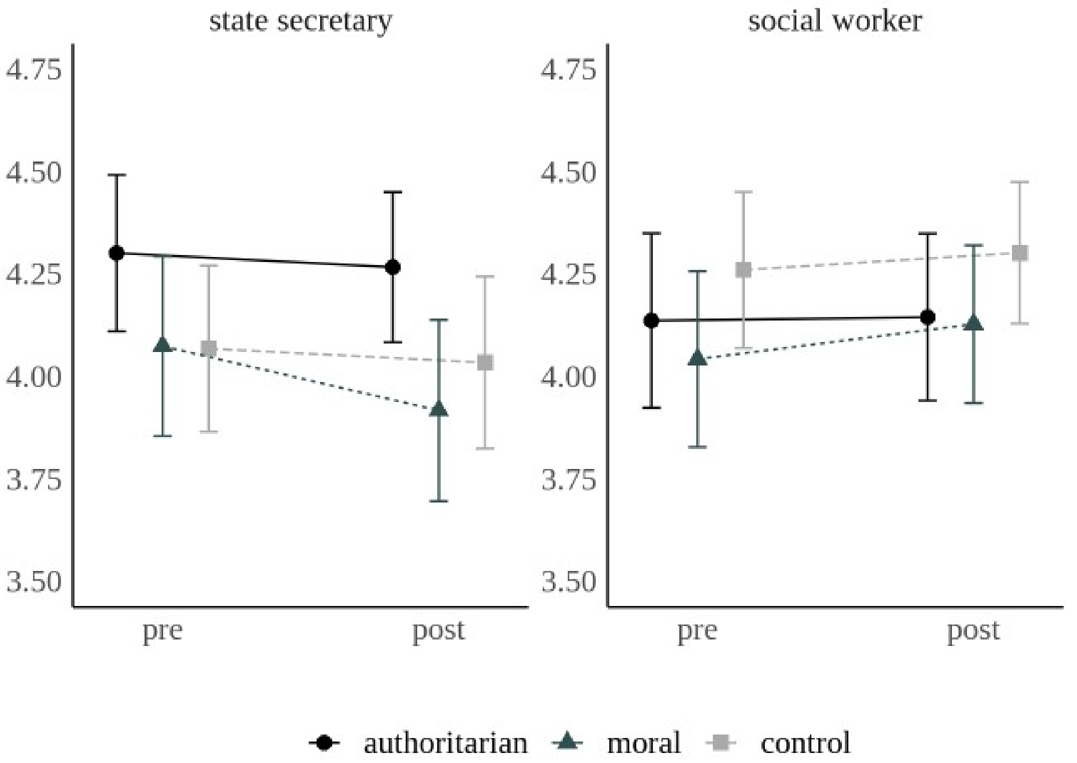

Item 4: There are only very limited reasons for me to leave the house: emergency care, important purchases, doctors visit, necessary work, meetings, exams, sport, physical activity. (*without correction of ceiling effect*)

Figure S1. Mean ratings (95% CI) in response to the single items, without correction of the ceiling effects *(continued)*

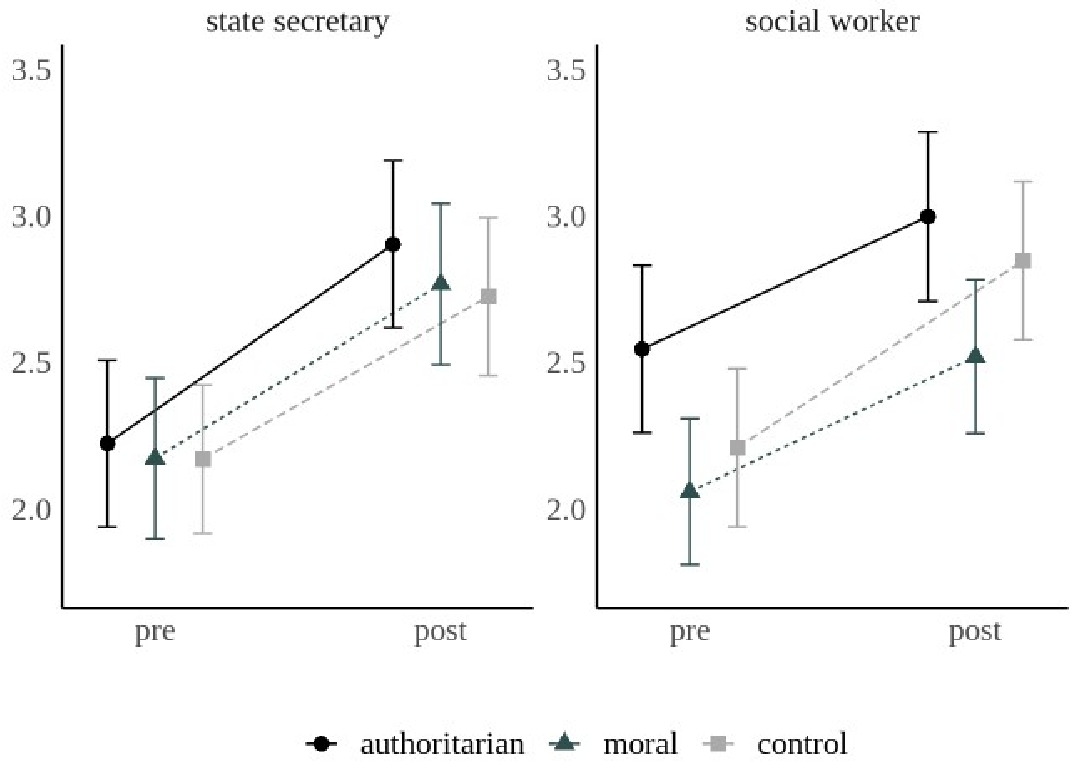

Item 5: I wear a protective mask when I am in other indoor rooms. (*without correction of ceiling effect*)

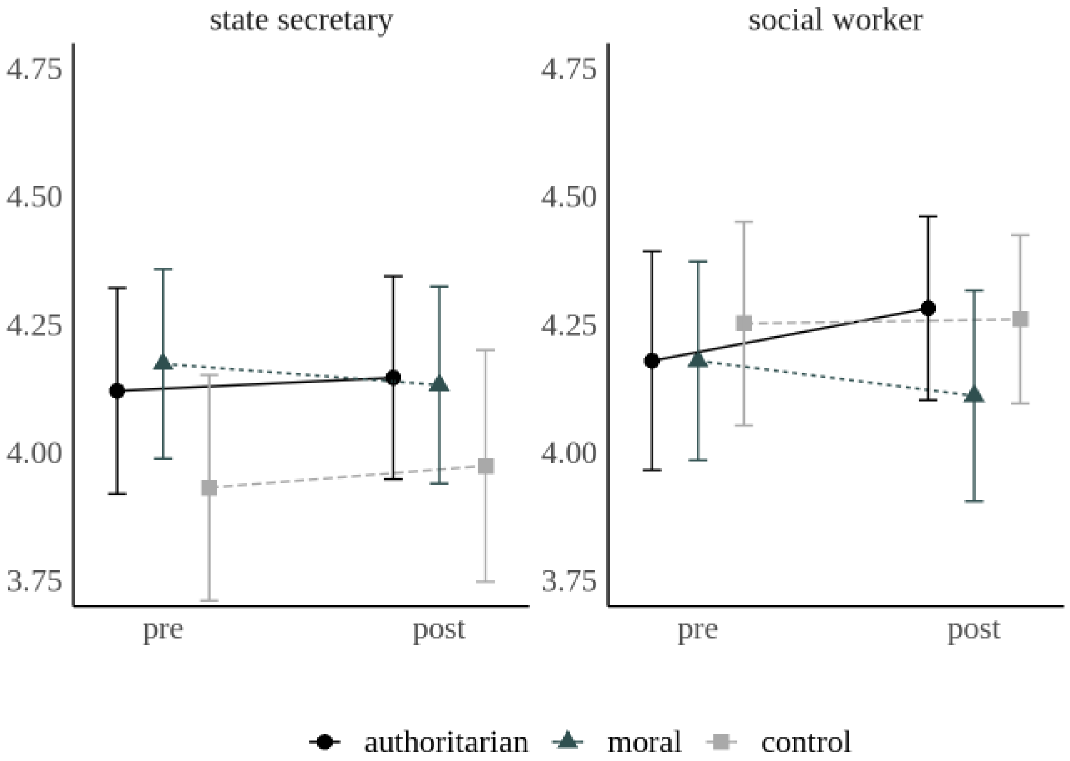

Item 6: For as long as schools and kindergartens are closed, I prevent my children from having any contacts, or I would do this if I had children. (*without correction of ceiling effect*)

Figure S1. Mean ratings (95% CI) in response to the single items, without correction of the ceiling effects *(continued)*

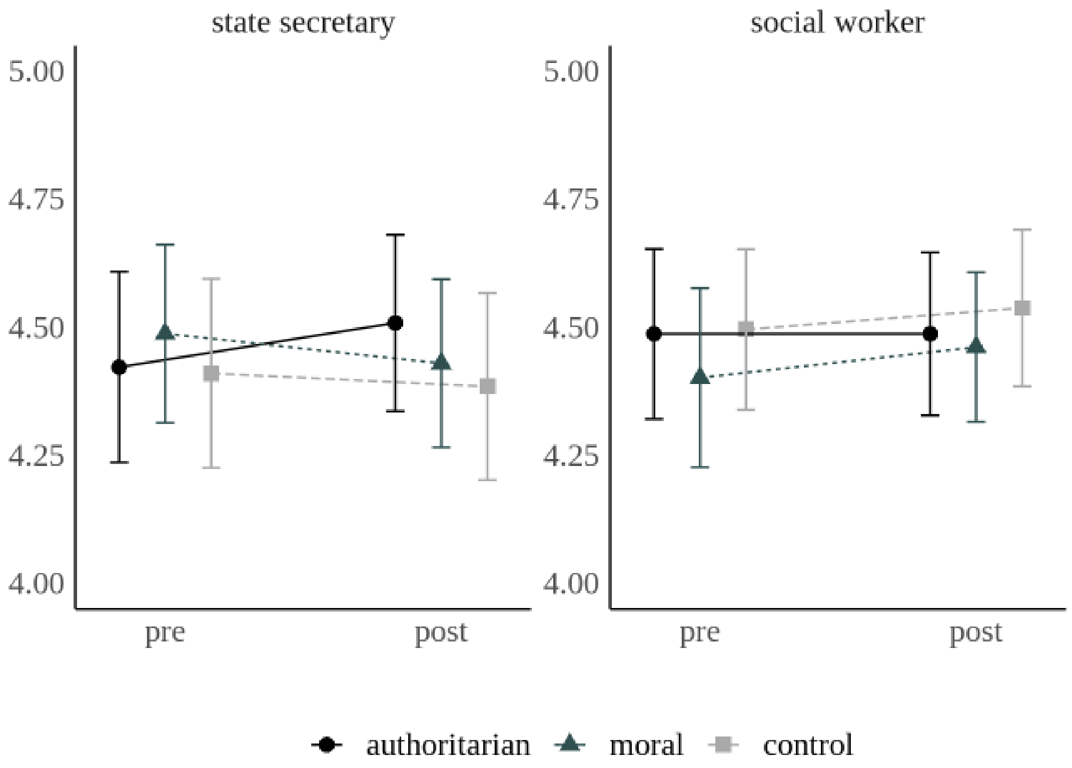

Item 7: I abstain from personal contact to older relatives and persons at risk. (*without correction of ceiling effect*)

Figure S1. Mean ratings (95% CI) in response to the single items, without correction of the ceiling effects. *(continued)*

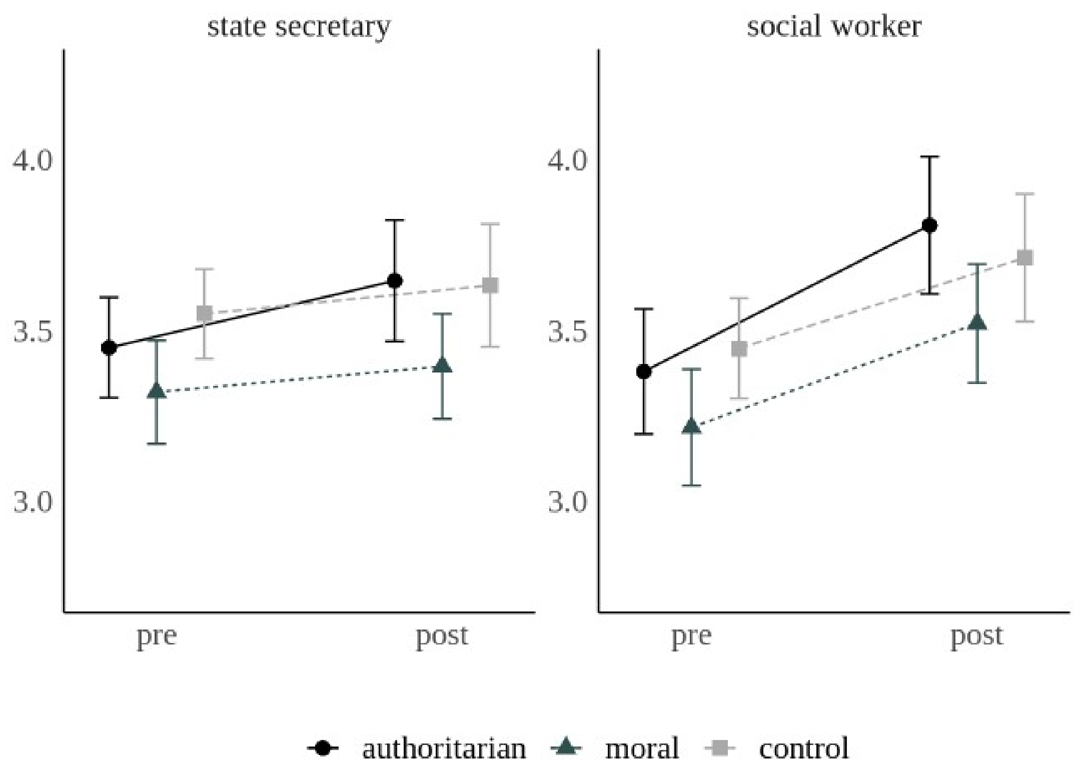

Item 1: I reduce contacts to other people outside the apartment to an absolute minimum. (*with* correction of ceiling effect)

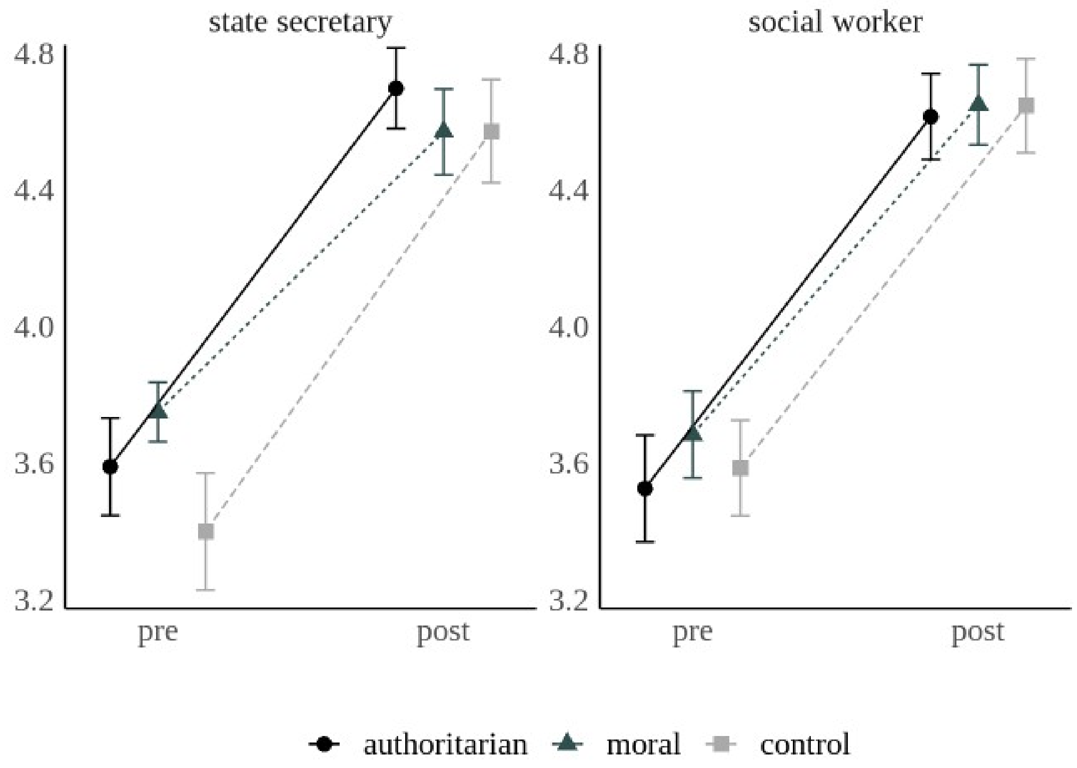

Item 2. I keep a minimum distance of 1.5 meter to other people in public wherever possible. (*with* correction of ceiling effect)

Figure S2. Mean ratings (95% CI) in response to the single items with correction of the ceiling effects.

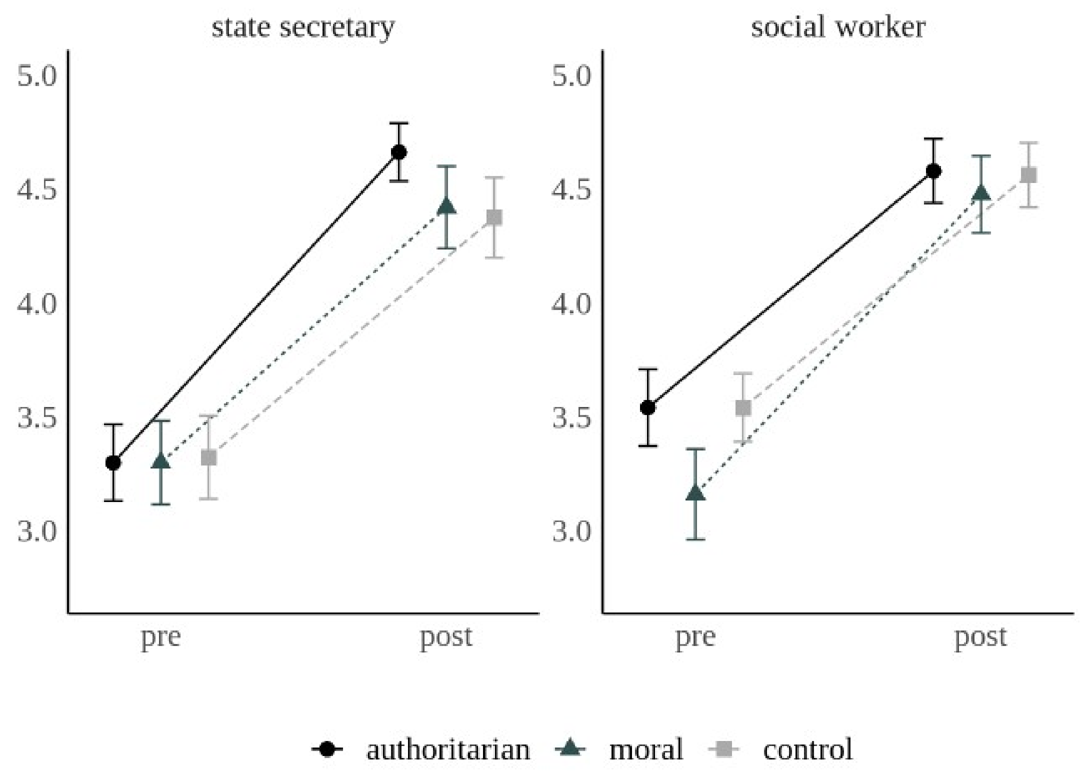

Item 3: I only spend time in public alone, with people of my household, or with one other person. (*with* correction of ceiling effect)

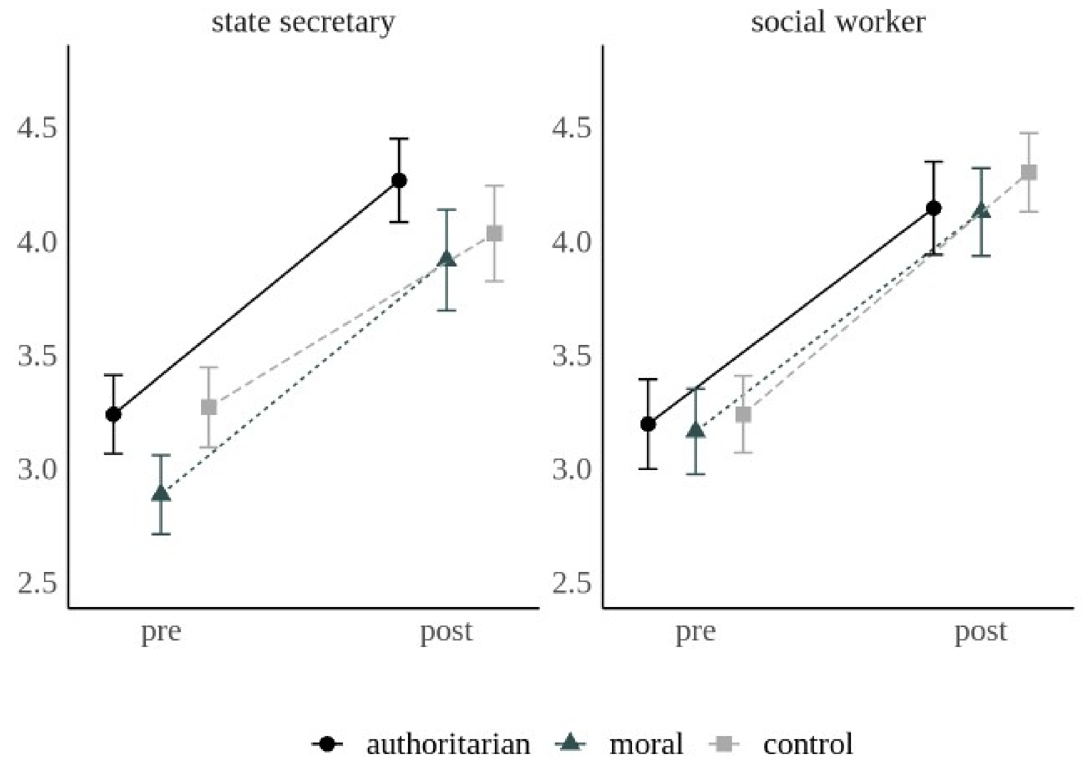

Item 4: There are only very limited reasons for me to leave the house: emergency care, important purchases, doctors visit, necessary work, meetings, exams, sport, physical activity. (*with* correction of ceiling effect)

Figure S2. Mean ratings (95% CI) in response to the single items with correction of the ceiling effects. *(continued)*

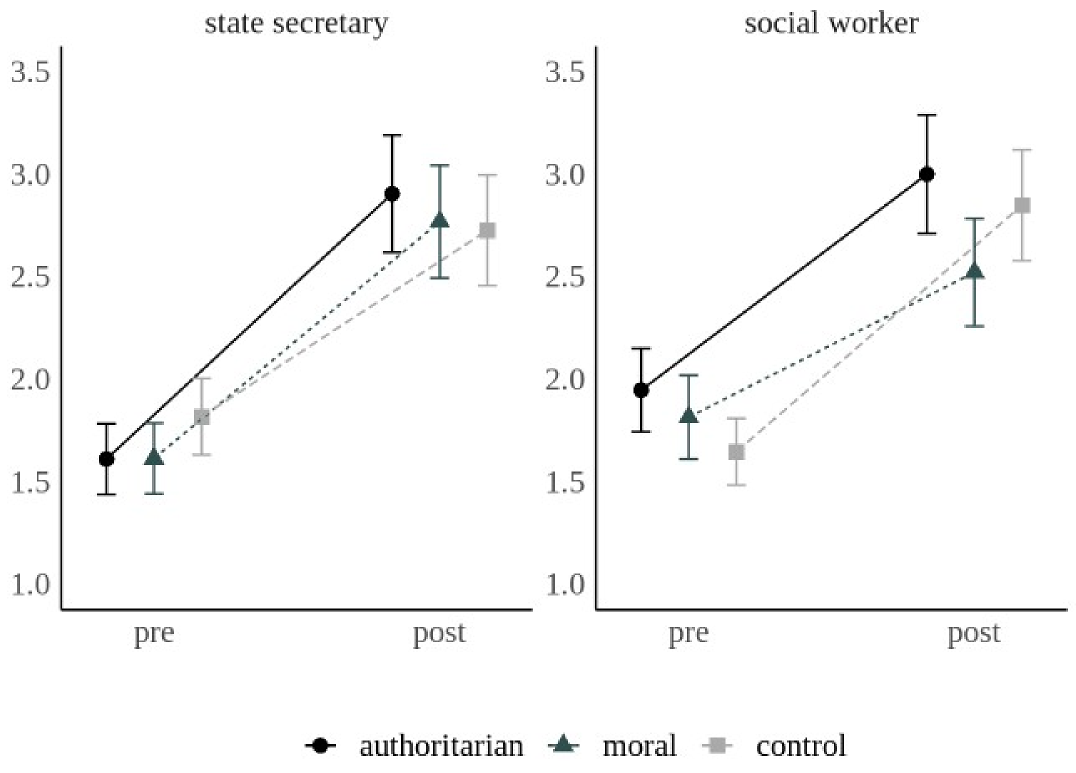

Item 5: I wear a protective mask when I am in other indoor rooms. (*with* correction of ceiling effect)

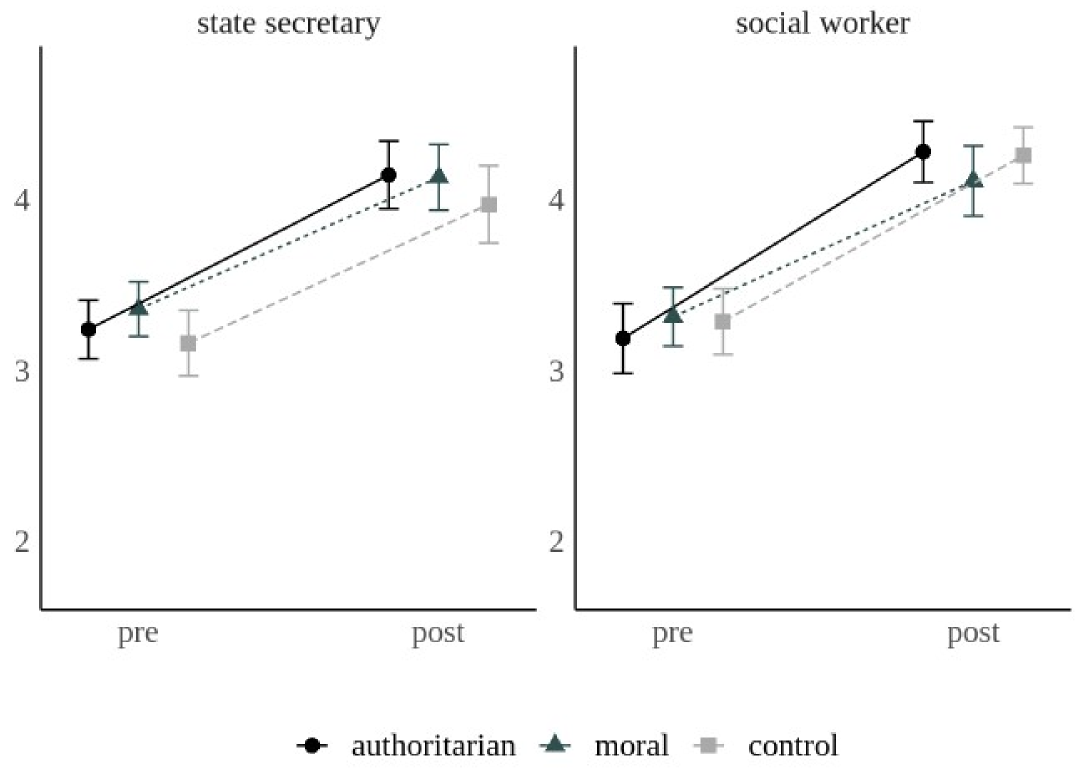

Item 6: For as long as schools and kindergartens are closed, I prevent my children from having any contacts, or I would do this if I had children. (*with* correction of ceiling effect)

Figure S2. Mean ratings (95% CI) in response to the single items with correction of the ceiling effects. *(continued)*

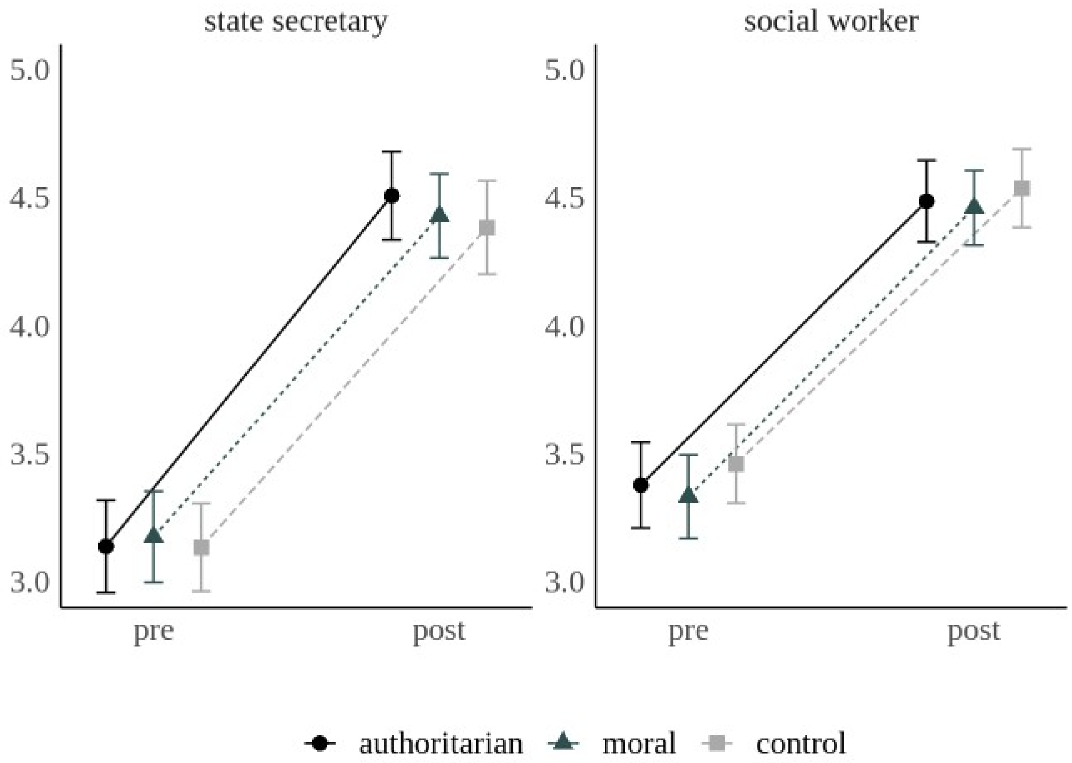

Item 7: I abstain from personal contact to older relatives and persons at risk. (*with* correction of ceiling effect)

Figure S2. Mean ratings (95% CI) in response to the single items with correction of the ceiling effects. *(continued)*

Table S5. Spearman correlations between trait autonomy and average bidirectional pre-post difference (averaged across all seven items) for the different senders (high and low authority) and message types, without correction of ceiling effects.

	high authority: state secretary	low authority: social worker
authoritarian/ controlling	*r* = -.02(*n* = 116), *p* = .81	*r* = -.06 (*n* = 117), *p* = .53
moral/ prosocial	*r* = -.07 (*n* = 121), *p* = .47	*r* = -.01 (*n* = 117), *p* =.94
control	*r* = -.05 (*n* = 117), *p* = .62	*r* = -.06(*n* = 119), *p* = .52

Note. p - values are Holm adjusted for multiple tests.

Table S6. Regression results using absolute pre-post differences (averaged across all seven items) as the criterion, without correction of ceiling effects (R^2 = .04, F(11, 695)= 2.72, p < 0.01).

Predictor	*b*	*b 95% CI*	*p*
(Intercept)	0.49	[-.24,1.23]	0.19
autonomy	-0.04	[-.25,0.16]	0.68
sender (social worker)	**1.23**	[0.20,2.27]	**0.02**
message (moral)	0.79	[-0.31,1.89]	0.16
message (control)	0.39	[-0.71,1.48]	0.49
autonomy x sender (social worker)	**-0.32**	[-0.61,-.04]	**0.03**
sender (social worker) x message (moral)	-1.15	[-2.66,0.36]	0.13
sender (social worker) x message (control)	-1.45	[-2.94,0.04]	0.06
autonomy x message (moral)	-0.21	[-.52,0.09]	0.16
autonomy x message (control)	-0.09	[-0.41,0.21]	0.53
autonomy x sender (social worker) x message (moral)	0.30	[-0.12,0.72]	0.16
autonomy x sender (social worker) x message (control)	0.38	[-0.04, 0.79]	0.07

Note. b represents unstandardized regression weights. Square brackets are used to enclose the lower and upper limits of a confidence interval.

Table S7. Spearman correlations between trait autonomy and average absolute pre-post difference (averaged across all seven items) for the different senders (high and low authority) and message types, with correction of ceiling effects.

	high authority: state secretary	low authority: social worker
authoritarian/ controlling	*r* = -.01 (*n* = 107), *p* = .91	*r* = -.16(*n* = 109), *p* = .09
moral/ prosocial	*r* = -.09 (*n* = 109), *p* = .35	***r* = -.19 (*n* = 113), *p* = .04**
control	*r* = .01 (*n* = 111), *p* = .93	*r* = .03(*n* = 107, *p* = .78

Note. p - values are Holm adjusted for multiple tests.

Table S8. Spearman correlations between trait autonomy and average bidirectional pre-post difference (averaged across all seven items) for the different senders (high and low authority) and message types, with correction of ceiling effects.

	high authority: state secretary	low authority: social worker
authoritarian	*r* = .04 (*n* = 107), *p* = .67	*r* = 0.02(*n* = 109), *p* = .85
moral/ prosocial	*r* = .06 (*n* = 109), *p* = .51	*r* = 0.01 (*n* = 113), *p* = .91
control	*r* = -.09 (*n* = 111), *p* = .36	*r* = .01(*n* = 107), *p* = .92

Note. p - values are Holm adjusted for multiple tests.

Table S9. Regression results using absolute pre-post differences (averaged across all seven items) as criterion, with correction of ceiling effects (R^2 = .01, F(11, 644) = 0.01, p = 0.75).

Predictor	*b*	*b* 95% CI	*p*
(Intercept)	0.54	[-0.76, 1.84]	0.41
autonomy	0.04	[-0.32, 0.40]	0.82
sender (social worker)	1.19	[-0.64, 3.01]	0.20
message (moral)	0.54	[-1.40, 2.49]	0.58
message (control)	-0.49	[-2.41, 1.44]	0.62
autonomy x sender (social worker)	-0.35	[-0.85, 0.16]	0.17
sender (social worker) x message (moral)	-0.85	[-3.51, 1.81]	0.53
sender (social worker) x message (control)	-0.66	[-3.32, 2.00]	0.63
autonomy x message (moral)	-0.18	[-0.72, 0.36]	0.51
autonomy x message (control)	0.12	[-0.42, 0.66]	0.66
autonomy x sender (social worker) x message (moral)	0.27	[-0.47, 1.00]	0.48
autonomy x sender (social worker) x message (control)	0.22	[-0.53, 0.96]	0.57

Note. b represents unstandardized regression weights. Square brackets are used to enclose the lower and upper limits of a confidence interval.
